# Engineering a nanostructured “super surface” with superhydrophobic and superkilling properties†

**DOI:** 10.1039/C5RA05206H

**Published:** 2015-05-12

**Authors:** Jafar Hasan, Shammy Raj, Lavendra Yadav, Kaushik Chatterjee

**Affiliations:** a Department of Materials Engineering, Indian Institute of Science, Bangalore, Karnataka, India 560012; b Centre for Nanoscience and Engineering, Indian Institute of Science, Bangalore, Karnataka, India 560012

## Abstract

We present a nanostructured “super surface” fabricated using a simple recipe based on deep reactive ion etching of a silicon wafer. The topography of the surface is inspired by the surface topographical features of dragonfly wings. The super surface is comprised of nanopillars 4 μm in height and 220 nm in diameter with random inter-pillar spacing. The surface exhibited superhydrophobicity with a static water contact angle of 154.0° and contact angle hysteresis of 8.3°. Bacterial studies revealed the bactericidal property of the surface against both gram negative (*Escherichia coli*) and gram positive (*Staphylococcus aureus*) strains through mechanical rupture of the cells by the sharp nanopillars. The cell viability on these nanostructured surfaces was nearly six-fold lower than on the unmodified silicon wafer. The nanostructured surface also killed mammalian cells (mouse osteoblasts) through mechanical rupture of the cell membrane. Thus, such nanostructured super surfaces could find applications for designing self-cleaning and anti-bacterial surfaces in diverse applications such as microfluidics, surgical instruments, pipelines and food packaging.

## 1 Introduction

“Super surfaces” with a range of exceptional properties are researched in many areas of science. There is particular interest in the field of medicine given the stringent demands of the healthcare sector. Different areas in healthcare such as biosensing,^1^ drug delivery,^2^ biomaterials and implants,^3^ therapeutics,^4^ and medical devices and instruments^5^ utilize the smartness of nanomaterials which exhibit self-healing,^6^ self-cleaning,^7^ superhydrophobic^8^ and antibacterial activity.^9^ Some of the most widely sought after properties include self-cleaning, superhydrophobicity and antibacterial activity.^10^–^13^ These properties could be either triggered by some external stimuli or may be inherently present as is observed in some nanostructured surfaces in nature. Surfaces that exhibit more than one such property can be called “super surfaces”.

Several strategies have been developed in an attempt to engineer surfaces with antibacterial activity to minimize biofouling.^9^,^12^,^13^ Antibacterial surfaces are classified as either antibiofouling or bactericidal surfaces based on the underlying mechanism. Antibiofouling surfaces repel the attachment and proliferation of the bacteria through unfavorable conditions present on the surface whereas bactericidal surfaces kill the bacterial cells by inactivating them mainly through chemical mechanisms.^9^ In order to eliminate the attached bacteria or inhibit the biofilm formation on the surfaces, new fabrication techniques have been devised and design improvements on the existing antibacterial surfaces have been proposed in the form of surface coating, surface chemical modification, and control of surface architecture.^9^,^14^ Surface coatings and chemical modifications have been further characterized into surface polymerization, functionalization and derivatization.^9^ However, the surface coatings or modifications have several significant drawbacks; firstly, the emergence of bacterial resistance against the antibiotics or antibacterial agents; secondly, the surface coatings or antibacterial agents can take a long time to leach from the surface; thirdly, the concentration of the surface coatings or antibiotics is limited and may not be maintained at optimum level to provide effective antibacterial activity over sustained periods of time; and, lastly, the durability of the surface may not be long enough to maintain the antibacterial activity.^9^,^15^–^19^ Emerging strains of antibiotic resistant superbugs pose a serious biomedical challenge. Discovery of new antibiotics are infrequent with a recent report after a span of nearly 30 years.^20^

Biomimicry can offer innovative and alternative solutions to overcome such challenges. The nano-architecture on surfaces of insect wings may offer an excellent platform for the design of super surfaces. Since both insect wings and prokaryotes are known to co-exist and evolve since millions of years, it is evident that the bactericidal insect wing nanostructures are able to consistently rupture the bacterial cells without encountering bacterial resistance in contrast to the chemical based antibacterial mechanisms. Two known nanostructured insect wing surfaces are those of cicada and dragonfly. The surface of such wings have been known to be self-cleaning, super-hydrophobic and antibacterial in addition to perhaps many as yet unexplored characteristics.^21^,^22^

There have been a few attempts to fabricate surfaces that mimic at least some of the unique properties of the insect wing surfaces.^11^,^23^ Whereas the antibacterial properties have been replicated in some recent studies, surfaces that are super-hydrophobic and yet bactericidal have not been accomplished.^21^,^24^ Moreover, the interactions of such a super surface with mammalian cells is not reported precluding its potential application in design of surfaces for biomedical implants.

The objective of this study was to engineer a super surface mimicking the superhydrophobic, self-cleaning and antibacterial characteristics of the surface of dragonfly wings through a facile fabrication technique. Deep reactive ion etching (DRIE) technique was utilized in this study because of its ease of use, high throughput and cost effectiveness when compared with other nanofabrication techniques. The water wettability and bacterial response to the surface was characterized. The cytocompatibility of the super surface generated was also evaluated.

## 2 Materials and methods

### 2.1 Fabrication of nanostructured surfaces

Nanostructured silicon surfaces were fabricated using the DRIE (SPTS) technique. A p-type boron-doped 100 mm diameter commercial silicon wafer with specific resistivity of 1–10 Ω cm, (100) oriented surface (GMS-India) was used as a substrate. The deposition cycle was performed with 185 sccm of C_4_F_8_ gas and the etch cycle was performed with SF_6_ and O_2_ gases at a flow rate ratio of 98 : 2. The power of the coil was set at 2000 W and 2150 W for the deposition and etch cycles, respectively, at time intervals of 2 s each. The pressure was maintained at 20 mT and 32 mT for the deposition and etch cycles, respectively.

### 2.2 Surface characterization

Surface morphology of the nanostructured and control surfaces were studied using a scanning electron microscope (SEM, Ultra55, Gemini) set at 7 kV with a secondary or in-lens detector. The EDX spectra were obtained at 15 kV using X-ray spectrometer (INCA suite v 4.15) interfaced with the SEM.

The static contact angles of three solvents namely ultrapure water (Sartorius Arium), glycerol (Sigma) and benzene (Sigma) were measured using a goniometer (OCA 15EC, Dataphysics) on the surface of the control silicon and nanostructured samples. Contact angle was measured 1 s after dispensing 1 µL of water on the sample. Three independent replicates were used for each sample. The advancing and receding water contact angle values were measured by dynamically adding and removing the volume of the droplet and keeping the solid/liquid interface area constant, respectively.^25^,^26^ The contact angle hysteresis was measured by subtracting the advancing and receding contact angle values. The surface free energy was calculated by the Lewis acid–base method or van Oss–Chaudhury–Good method which employ the measurements of the static contact angles of the three solvents.^27^,^28^

### 2.3 Bacterial response

Bacterial strains of *Escherichia coli* (DH5A) and *Staphylococcus aureus* (ATCC 25923) were used as the model gram negative and gram positive bacteria, respectively. Cells were grown in 100 mL of sterile nutrient broth (HiMedia) overnight at 200 rpm and 37 °C. Bacterial cultures were cultured on nutrient agar (HiMedia). The cells were collected at the logarithmic stage of growth and the concentration of the suspensions was adjusted to OD_600_ (optical density at 600 nm) value of 0.25 in 25 mM phosphate buffered saline (PBS, Sigma) solution before incubation with the nanostructured surface measuring 0.5 cm x 0.5 cm in area. The as-received Si wafer without nanostructures of the same dimension was used as the control. In addition, a well without a Si wafer served as an additional control. The nanostructured and control surfaces were immersed in 1 mL of the bacterial suspension in a 24-well plate. The bacterial cell viability was assessed by measuring the absorbance values at OD_600_ after 1 min, 5 min, 10 min, 30 min, 1 h, 3 h and 6 h. The growth of the adhered cells on the nanostructured and control silicon surfaces was identified at different time intervals of 6 h, 10 h, 24 h and 30 h by measuring the absorbance values at OD_600_ in triplicates.

In addition, 100 µl of the cell suspensions after 1 h of incubation with the nanostructured and control surfaces were taken and diluted ten folds. Each of the diluted suspensions was spread on to three nutrient agar plates. Resulting colonies were then counted after 24 h of incubation at 37 °C, and the number of colony forming units per mL was calculated. To assess the morphology of the adherent bacterial cells after 1 h, the nanostructured and control surfaces were washed with fresh PBS and fixed with 2.5% glutaraldehyde for 30 min. The samples were sputtered with gold prior to imaging by SEM.

Viability of the adhered bacterial cells was determined by counting of cells stained with the LIVE/DEAD® BacLight™ Bacterial Viability kit (Molecular Probes, Invitrogen) at different incubation periods of 5 min, 30 min, 60 min and 180 min. Adherent cells were stained using 3.3 mM SYTO 9 and 20 mM propidium iodide for 15 min and imaged with an inverted fluorescence microscope (Leica) in both the green and red channels. The fraction of viable cells was determined by counting cells stained green and red from three images of at least three independent replicates.

### 2.4 Osteoblast response

Biological response to the samples was evaluated *in vitro* using the MC3T3-E1 subclone 4 mouse cell line (ATCC), as reported earlier.^29^ The cells were cultured in α-minimum essential medium (α-MEM) supplemented with a 10% (v/v) of fetal bovine serum (FBS, Gibco, Life Technologies).^30^ Trypsin–EDTA was used for passaging and the cells were further sub-cultured. Passage 3 cells were used for all the reported studies herein. All silicon samples were sterilized by immersing in ethanol for 30 min followed by exposure to UV for 1 h prior to seeding the cells. Samples (0.25 mm × 0.25 mm) were placed individually in a 96-well tissue culture polystyrene (TCPS) plate. 200 μL of cell suspension containing 5 × 10^3^ cells was added to each well. Cell attachment and proliferation were imaged 3 days after seeding. Fluorescently labeled cells were imaged to characterize cell morphology. Four replicates of each sample were used: two replicates for SEM and two for fluorescent imaging.

For SEM imaging, adherent cells on the control and nanostructured surfaces were washed with PBS solution and fixed with 3.7% formaldehyde 37 °C for 15 min. The samples were then mounted and gold sputtered prior to SEM imaging. For fluorescence imaging, cells were fixed using 3.7% formaldehyde at 37 °C for 15 min. The cells were subsequently permeabilized with 0.2% Triton X (Sigma Aldrich). Actin filaments were stained using 25 µg mL^−1^ Alexa fluor 546 (Invitrogen) at 37 °C for 15 min. Cell nuclei were stained using 0.2 µg mL^−1^ DAPI (Invitrogen) at 37 °C for 5 min. Stained cells were imaged with an inverted fluorescence microscope (Olympus). For viability analysis, the LIVE/DEAD® Viability/Cytotoxicity Assay Kit (Molecular Probes, Invitrogen) was utilized as described above for bacterial cells. Adherent cells were stained using 2 µM Calcein and 4 µM ethidium homodimer-1 at 37 °C for 30 min in order to determine the fraction of viable cells from fluorescence images (Olympus).

## 3 Results and discussion

**Fig. 1:**
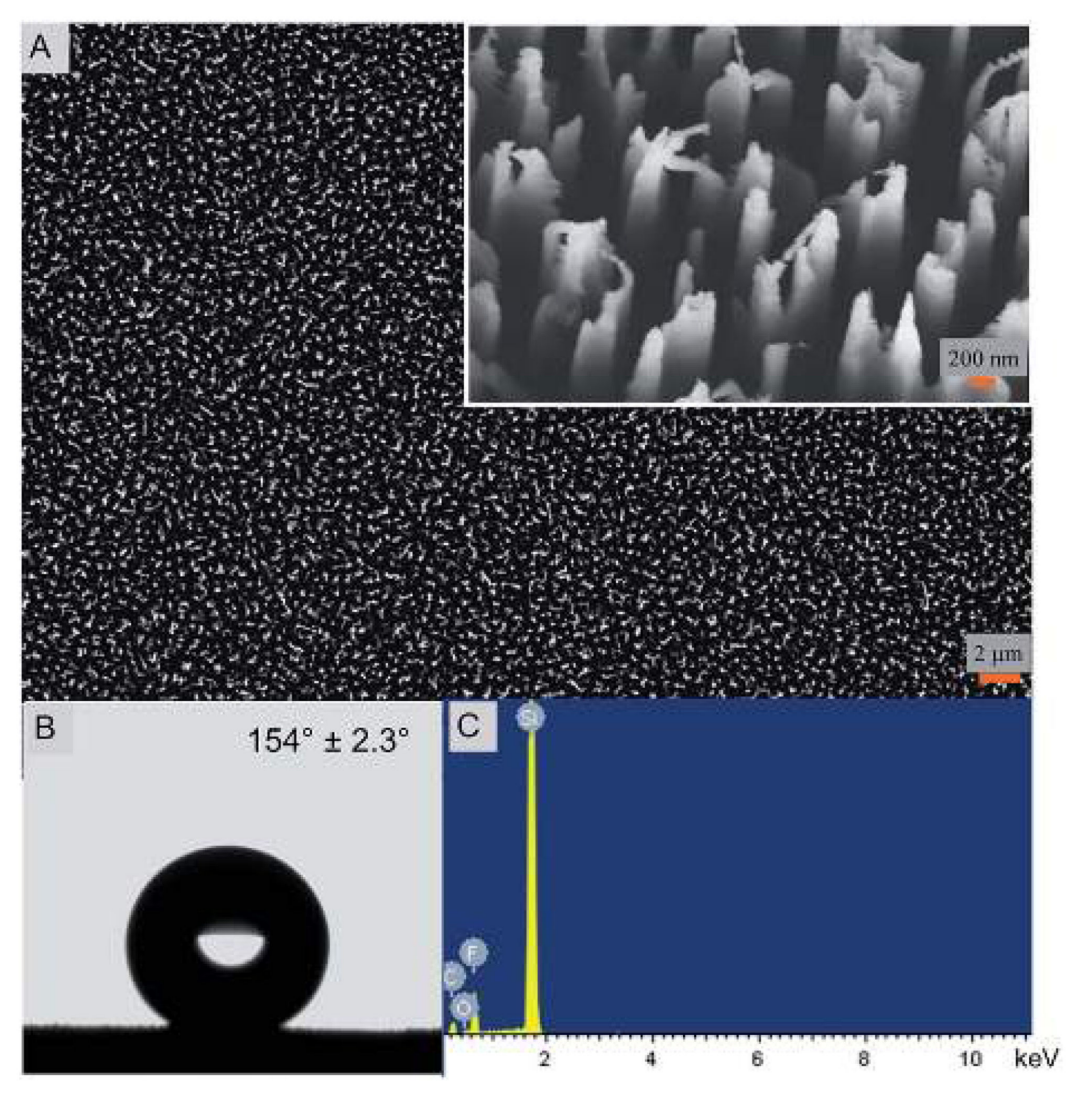
(A) Electron micrograph of the nanostructured silicon super surface fabricated using DRIE. The inset presents the tilted view of the nanopillars. (B) The super surface displayed a static water contact angle of 154° indicating superhydrophobicity. (C) The EDX spectra of the fabricated silicon showing the presence of elements.

The nanostructured silicon surface (Fig. 1) was fabricated by a simple DRIE technique or the Bosch process. In the last decade, the DRIE process has evolved as one of the widely used fabrication technique in the micro-electro mechanical system (MEMS) industry.^31^ It is mainly used to produce high-aspectratio silicon surfaces for designing the semiconductors and photovoltaics materials.^32^ Interestingly, the DRIE processes have also been used in some biological applications very recently.^21^,^33^ Here, the silicon nanostructures are 4 μm tall and 220 nm in diameter with extremely sharp peaks. The width of the peak varies between 10–20 nm. The etched nanopillars are not smooth on its walls and exhibit a usual scalloping pattern because of the consecutive etching and passivation steps using SF_6_ and C_4_F_8_ gases, respectively (Fig. 1A).^34^ Due to the high aspect ratios, the etched silicon sometimes is antireflective and turns black in color after the etching and is therefore termed as ‘black silicon’. Black silicon has been used for solar cell applications.^35^

The nanostructured surface is superhydrophobic in nature with a static water contact angle of 154.0° ± 2.3° (Fig. 1B). The contact angle hysteresis (CAH) value of the nanostructured surface is 8.3° with advancing and receding values at 154.3° and 146.0°, respectively. This indicates that the superhydrophobic surface has a low adhesion and is thereby self-cleaning. The CAH is closely related to the roll-off angle and CAH values less than 10.0° are termed self-cleaning in nature.^10^,^11^ The surface free energy, calculated by the van Oss–Chaudhury–Good method, was calculated to be 18.8 mJ m^−2^ (Table 1). The surface energy value is significantly lower in comparison to the control surface and other reported surfaces further confirming the low adhesive nature of the super surface prepared herein.^36^–^39^

In order to understand the chemical composition of the super surface, EDX analysis of the nanostructured surface was performed. The presence of fluorine, oxygen and carbon along with silicon is evident that may be attributed to the use of the etching and passivating gases during fabrication (Fig. 1C and ESI, Table S1†). The silicon control surface exhibited only the presence of silicon and carbon (ESI, Fig. S1 and Table S2†).

It is widely believed that it is difficult to achieve superhydrophobicity and self-cleaning property due to nanoscale topography alone and hierarchical roughness is essential to impart these properties.^40^,^41^ However, there are a few natural surfaces that exhibit superhydrophobicity and self-cleaning ability based only on their nanotopography such as insect wings.^21^,^22^,^42^–^44^ Recently, the self-cleaning property of a nanostructured surface has been demonstrated by the jumping mechanism rather than the usual sliding or tilt angle measurement of the water droplet.^44^ Here, due to the superhydrophobicity and low adhesion, the surface exhibits a similar behavior as the cicada wing^44^ as the water droplet was unable to adhere and rolled off the surface (ESI, Video S1†). The superhydrophobicity of the nanostructured surface is also facilitated by the C_4_F_8_ gas discharges during the fabrication stage that deposits a hydrophobic Teflon-like (polytetrafluoroethylene, PTFE) passivation layer on the silicon material^45^,^46^ as confirmed by the EDAX measurements (Fig. 1C). Thus, a combination of chemical composition of the outer layer and the nanostructured topography imparts superhydrophobicity and self-cleaning ability to this super surface in contrast to other such surfaces based on multi-scale topography.

**Fig. 2:**
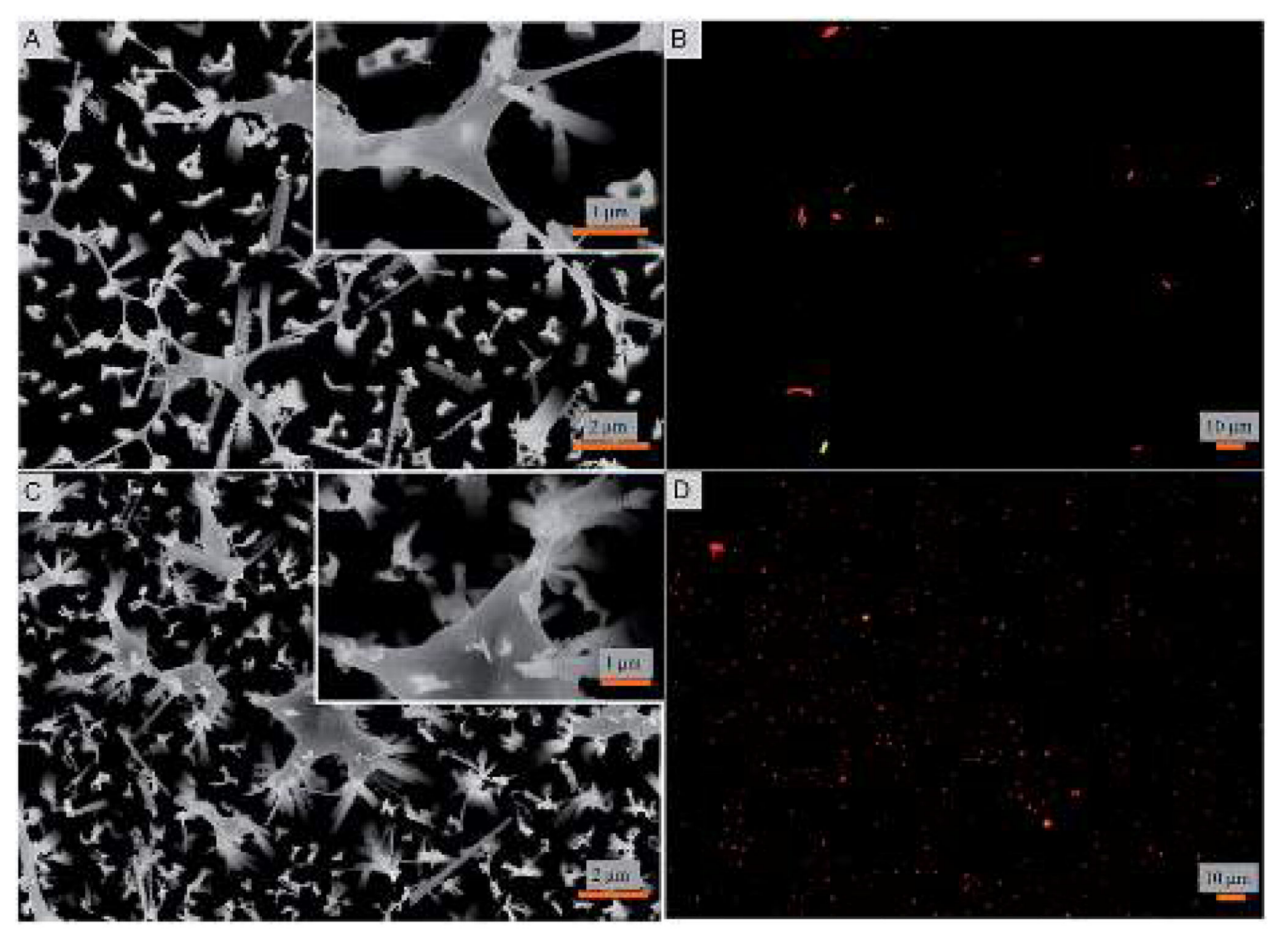
(A and C) Scanning electron micrographs and (B and D) fluorescent microscopic images of bacterial attachment on the fabricated super surfaces. *E. coli* (A and B) and *S. aureus* (C and D) cells are shown to be ruptured by the nanopillars. The fluorescent micrographs display the viable (green) and non-viable (red) cells.

Bacterial attachment studies were performed to test if the nanostructured superhydrophobic surfaces exhibit any antibacterial activity. Viability of *E. coli* and *S. aureus* were tested as model Gram negative and Gram positive strains on contact with the nanostructured surfaces. The bacterial cell response was assessed using a number of measures. The fate of adhered cells was evaluated by electron and fluorescence microscopy. Independently, the fate of cells in suspension in contact with the nanostructured surface was also measured. SEM images reveal that the nanopillars stretch the bacterial cells of both strains to the limit until they are ruptured (Fig. 2). A similar observation was reported for cicada wings that stretch the membrane of Gram-negative rod shaped cells.^22^,^47^ Interestingly, herein even the thicker membranous coccoid-shaped cells are seen to be ruptured in the same manner of stretching (Fig. 2B). It seems that the spherical shaped cells exhibit morphological deformations in the same manner as they are ruptured by the previously studied black silicon material.^21^ However, here the stretching of the membrane and the cellular disruption is to a markedly greater extent and evident in both the cellular strains (Fig. 2), when compared from the black silicon material reported previously. It appears that the cells whilst trying to adjust in the nanostructured surface are captured by the individual nanopins as the nanopins hold on to the cellular membrane. As the cells further move due to their motile behavior, the cells stretch themselves while few boundaries are still held by the nanopins. Upon reaching the limit of stretching, the cells are no longer able to survive and are therefore ruptured and killed. The intact bacterial cells attached to smooth control surfaces are shown in ESI (Fig. S2 and S3†) exhibiting intact morphology. These results are consistent with the previous bactericidal reports of wings and wing inspired studies.^21^,^22^,^47^

**Fig. 3:**
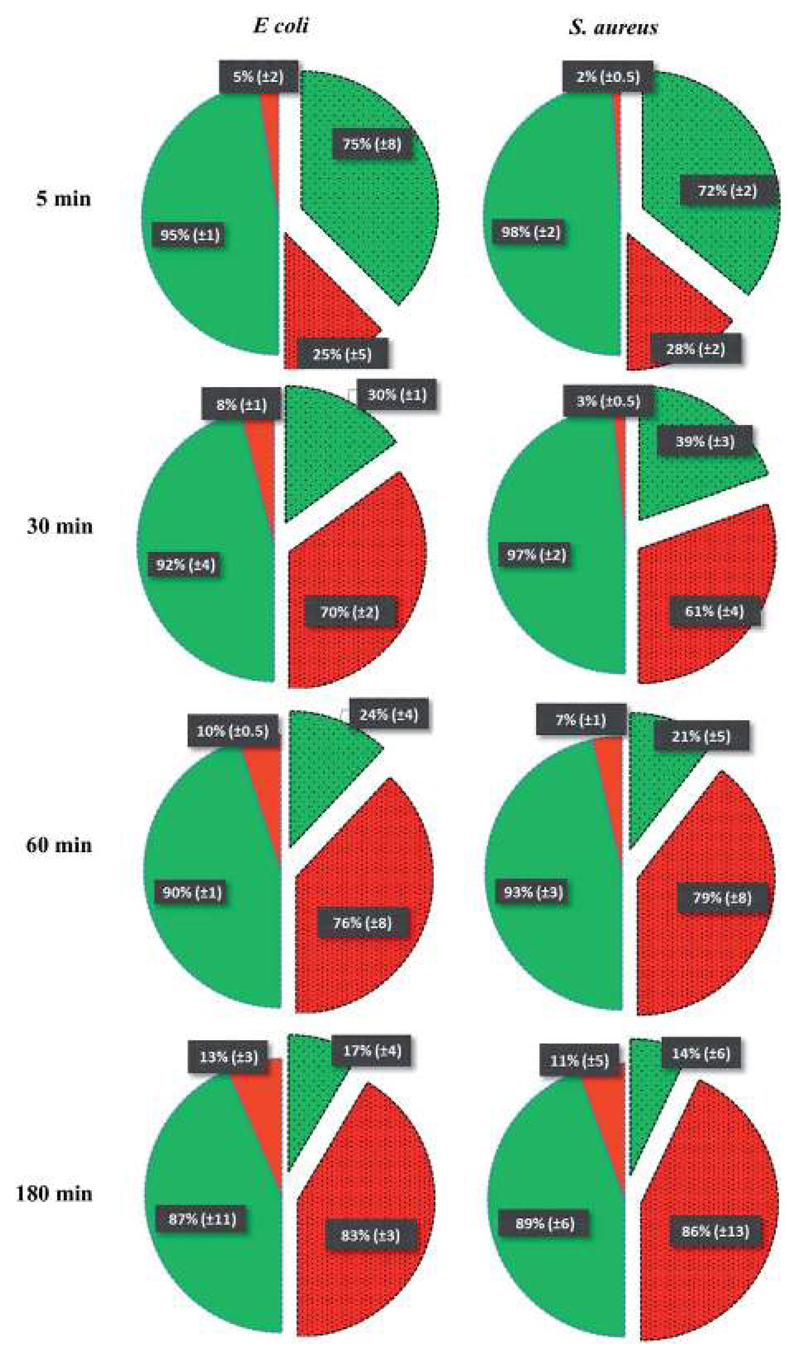
The percentage representation of non-viable and viable cells incubated over different time intervals is represented by a pie-chart on the control (non-patterned pies) and nanostructured (patterned and segmented pies) surfaces.

To test the killing efficiency of the nanostructured surfaces, live and dead adhered cells were counted using the fluorescent microscopy images. Of the total number of the bacterial strains attached on the nanostructured surfaces, 86% of *S. aureus* and 83% of *E. coli* cells were found to be non-viable after 3 hours of incubation. In contrast, on the control surfaces, cell viability was high with the non-viable fraction of only 11% of *S. aureus* and 13% of *E. coli* strains (Fig. 3). The over six-fold increase in the killing rate demonstrates the super killing nature of the nanostructured surfaces compared to the control smooth surfaces. However, this anti-bacterial activity was observed to be time dependent with only a quarter of the cells killed in 5 min. This fraction increased to >60% by 30 min. Thereafter, the fraction of dead cells increased with time to >80% by 3 h. Thus, approximately 30 min of contact with the super surface is required to induce inactivity of a substantial fraction of bacterial cells.

In order to further confirm the inactivity of the bacterial cells on the nanostructured surfaces, the absorbance values of the cell suspension was measured after incubation of 1 min, 5 min, 10 min, 30 min, 1 h, 3 h and 5 h. The suspensions for either strains exhibit lower absorbance compared to the smooth silicon surface control and negative control (no surface) suggesting reduced cell viability (ESI, Fig. S4 and S5†). The growth of the bacterial cells was also examined over a period of 30 h when the adhered cells on the nanostructured and control surface were allowed to grow in the nutrient media. The growth of both the strains in the nutrient medium containing the control surface was higher than the growth on the nanostructured surface further corroborating the antibacterial property of the nanostructured surface (ESI, Fig. S6 and S7†). The inactivity of bacterial cells were also assayed by the plate count method. The number of colonies of both the cell types in suspension were significantly reduced on the nanostructured surfaces compared to the silicon surfaces (ESI Fig. S8†). Thus, all measures of bacterial response confirmed the excellent bactericidal property of the super surface fabricated herein mediated by mechanical rupture of the cell membrane. This is in sharp contrast to other strategies based on the use of micro/nano structured surfaces that aim to reduce bacterial infection by minimizing cell adhesion.^48^,^49^

**Fig. 4:**
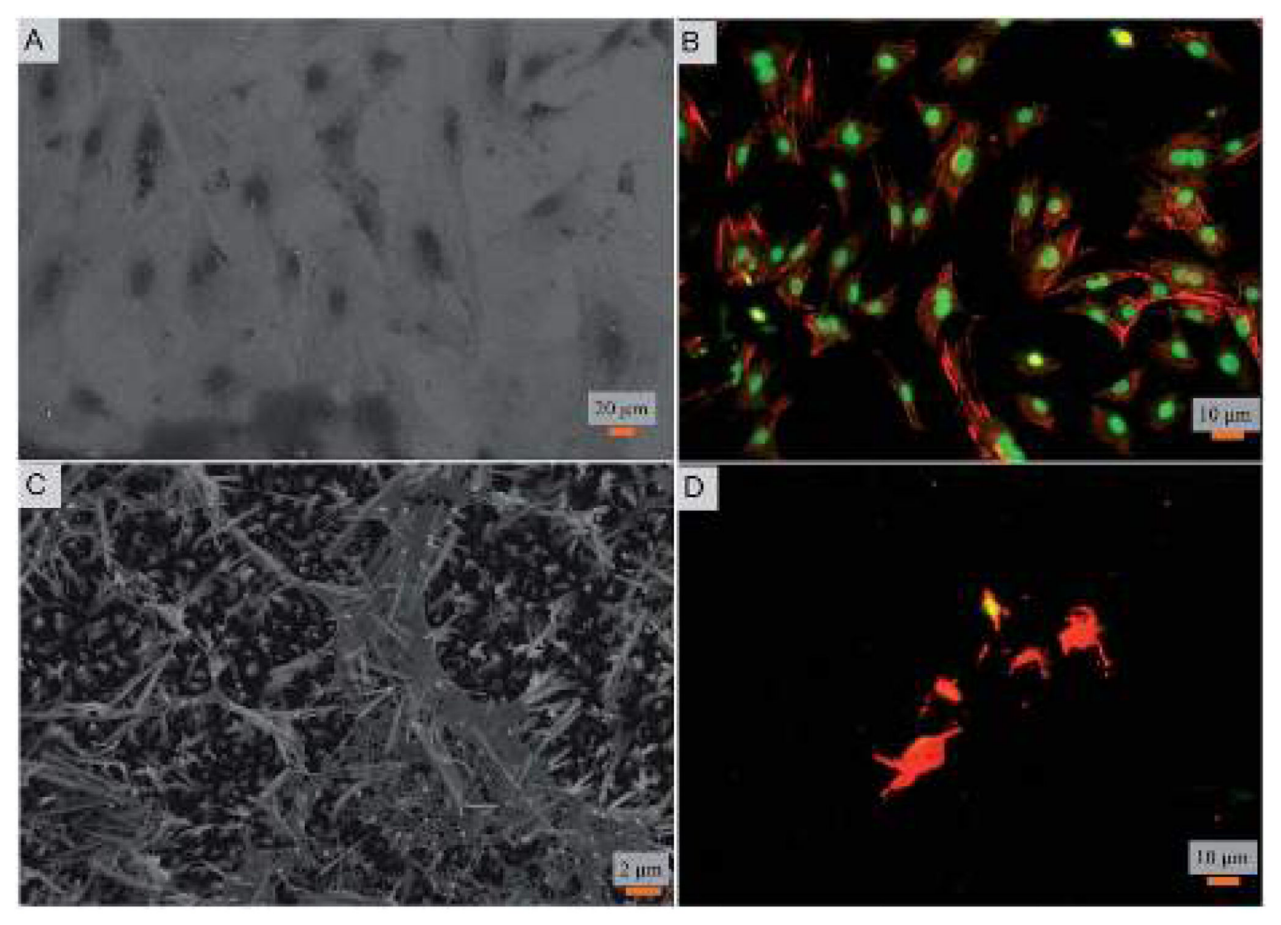
(A) Scanning micrograph and (B) fluorescent (red labeled actin and green labeled DAPI) images of mouse osteoblasts on the control surfaces. (C) Scanning micrograph and (D) fluorescent (red for F-actin and green for nucleus) images of osteoblasts on the fabricated nanostructured surfaces.

**Fig. 5:**
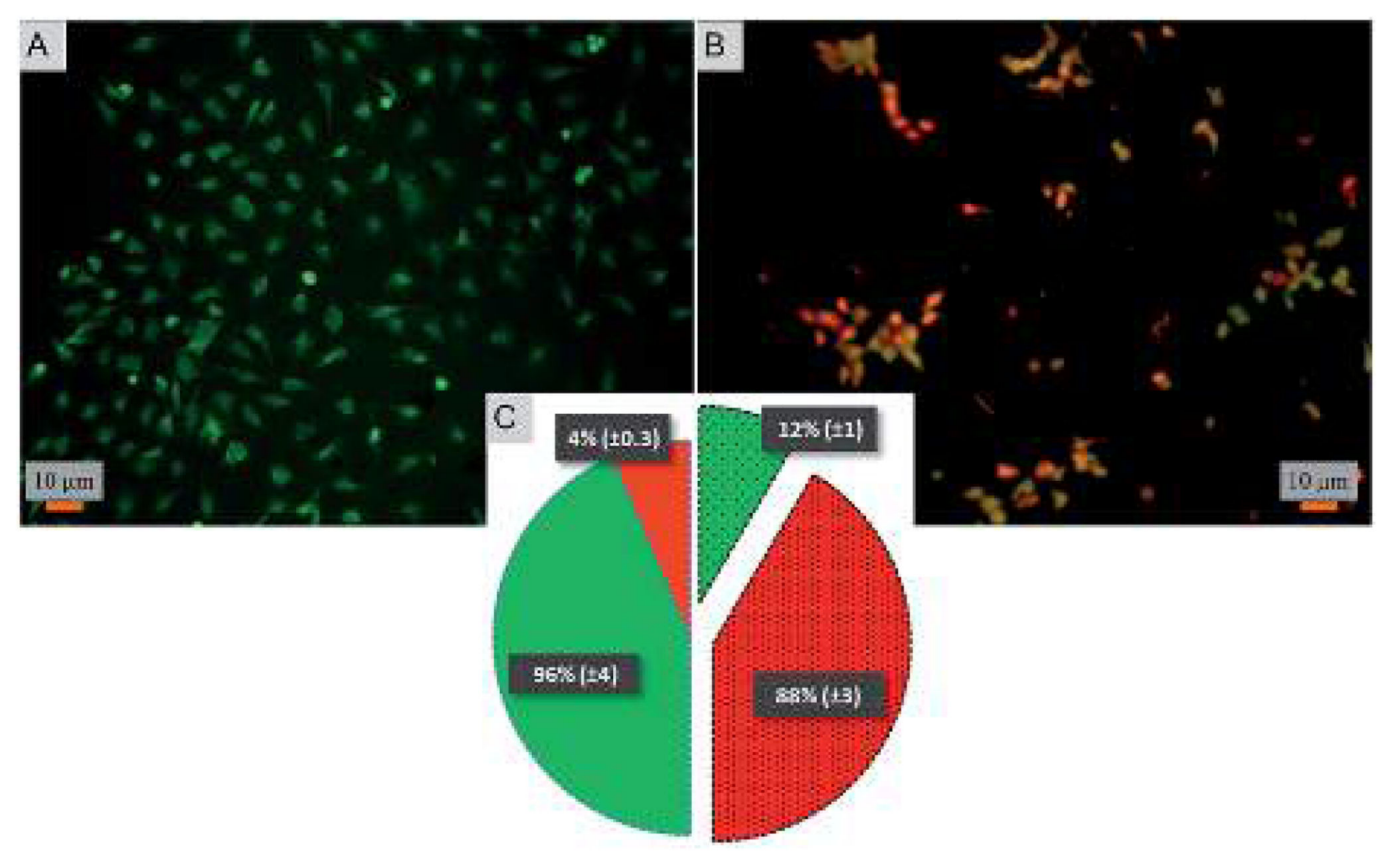
Viability assay of osteoblasts on (A) control and (B) nanostructured surface. Viable cells are stained green while non-viable cells are stained red. (C) The percentage distribution of viable and nonviable cells on the control (non-patterned pies) and nanostructured (patterned and segmented pies) surfaces.

Toward exploring the potential utility of such a super surfaces for use on biomedical implants such as prosthetic joints, stents, and fracture fixation devices, *etc.*, the cytocompatibility of the surface was evaluated. The viability and morphology of mouse osteoblasts on the surfaces was characterized. SEM and fluorescent micrographs indicate mechanical disruption of the membrane and low viability of cells on the super surface (Fig. 4 and 5). On the control surfaces, the cells were well spread and viable unlike the nanostructured surface where the excessive stretching of the cells on the nanopillars eventually led to the cell rupture and death. Live/dead staining confirmed that cell viability was only 12% on the nanostructured surfaces (Fig. 5).

Few reports have shown that different kinds of mammalian cells can proliferate on vertical nanostructured surfaces.^50^–^52^ However, the nanostructures on these reports differ in the aspect ratio and the close-packing when compared to this study. Moreover the bacterial attachment experiments were not reported. In a recent study, cicada wing inspired nanowired surfaces were fabricated that discriminate between the bacterial cells and osteoblasts such that the bacterial cells were significantly reduced whereas the osteoblasts were shown to adhere and proliferate.^24^ In the reported study, the nano-features were not vertically aligned when compared with the natural cicada wing nanoarchitecture.^22^ Also, because of the brush type or individual nanowires, the surface was unable to lyse the mammalian cells but in due course the antibacterial efficiency was also compromised as the nanowires were unable to pierce the Gram positive *S. aureus* cells. Nevertheless, there is a growing interest in the fabrication of vertical nanostructures for biological applications^53^ and leading to optimal designs for super surfaces.

Taken together, the findings of this study suggest that the super surface prepared herein exhibits superhydrophobicity and self-cleaning properties along with super killing properties for both bacterial and mammalian cells. Thus, such a super surface will be well suited for a variety of anti-bacterial surfaces but not on biomedical implants requiring intimate contact with mammalian cells *in vivo*. However in the field of medicine, the potential of such super surfaces extends to surgical instruments, tubing, and diagnostics tools, *etc.* to maintain ultraclean, dry and aseptic conditions.^5^ In addition, such super surfaces can be utilized for food packaging and microfluidics where contamination, dust, dirt, moisture and infections are serious concerns. This study proposes the functionality and potential utility of such nanostructured surfaces. Further work is required to develop strategies to engineer such super surfaces on materials used to fabricate instruments and products for biomedical use.

## 4 Conclusion

A super surface was prepared in this study that is inspired by the nanostructures present on insect wing surfaces. The super surface is shown to exhibit self-cleaning and superhydrophobic properties in nature. It is a super killer surface that kills both bacterial and mammalian cells by mechanical rupture. Such a super surface may find use in different applications requiring antifouling and self-cleaning properties.

## Supplementary Material

† Electronic supplementary information (ESI) available. See DOI: 10.1039/c5ra05206h

SI

SI movie

## Figures and Tables

**Table 1:** Static contact angle and surface energy values of the control (silicon wafer) and nanostructured surface

	Contact angle^a^ (degrees)			Surface energy components^b^ (mJ m^−2^)				
Substrate	*θ*_W_	*θ*_G_	*θ*_B_	*γ*^TOT^	*γ*^LW^	*γ*^AB^	*γ*^+^	*γ*^−^
Control surface	74.7 ± 1.8	66.8 ± 1.2	16.0 ± 0.8	33.2	26.0	7.1	1.2	10.6
Nanostructured surface	154.0 ± 2.3	139 ± 0.1	71.0 ± 1.8	18.8	14.8	3.9	2.6	1.5

a *θ*_W_, *θ*_G_ and *θ*_B_: water, glycerol and benzene contact angles, respectively.

b Surface energy components: Lifshitz–van der Waals (*γ*^LW^), acid/base (*γ*^AB^), electron acceptor (*γ*^+^) and electron donor (*γ*^−^) components.
