# Supplementary material for: Engineering a nanostructured “super surface” with superhydrophobic and superkilling properties†
Source: RSC Adv. Author manuscript; Available in PMC 2017 Oct 24. (PMC5654505; doi:10.1039/C5RA05206H)
Supplement: SI [file NIHMS74547-supplement-SI.pdf]

Electronic Supplementary Material (ESI) for RSC Advances.
This journal is © The Royal Society of Chemistry 2015

*Supplementary Information for*

# Engineering a Nanostructured "Super Surface" with Superhydrophobic and Superkilling Properties

Jafar Hasan[a], Shammy Raj[a], Lavendra Yadav[b], Kaushik Chatterjee[a]*

[a]Department of Materials Engineering and [b]Centre for Nanoscience and Engineering

Indian Institute of Science, Bangalore, Karnataka, India 560012

* *author to whom all correspondence should be addressed*

Email: kchatterjee@materials.iisc.ernet.in

Tel: +91-80-22933408

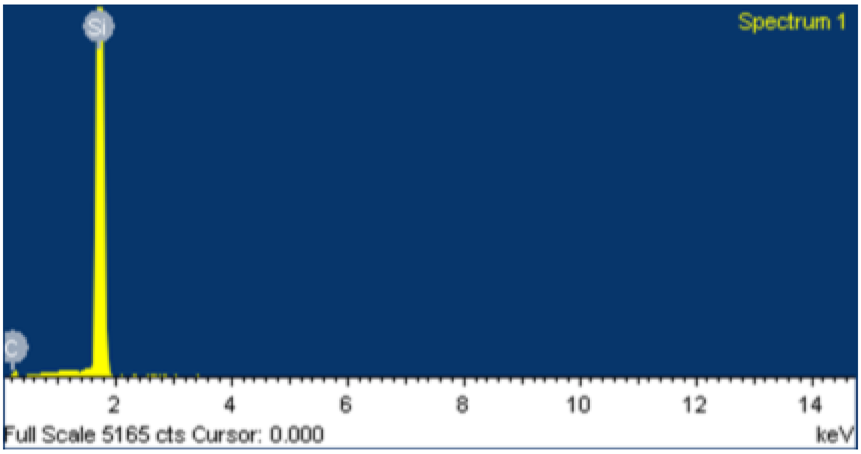


Figure S1. EDX spectra of the control (Silicon wafer) surface.

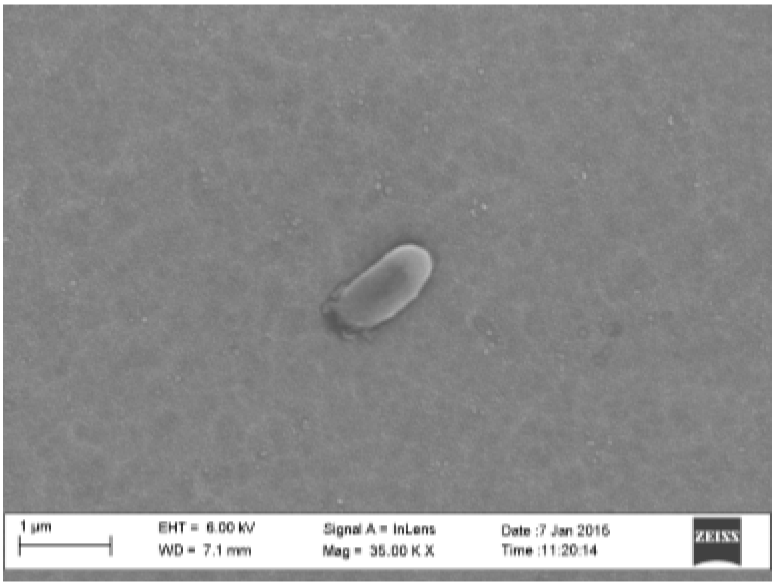


Figure S2. SEM image of an intact *E. coli* cell on a control (Silicon wafer) surface.

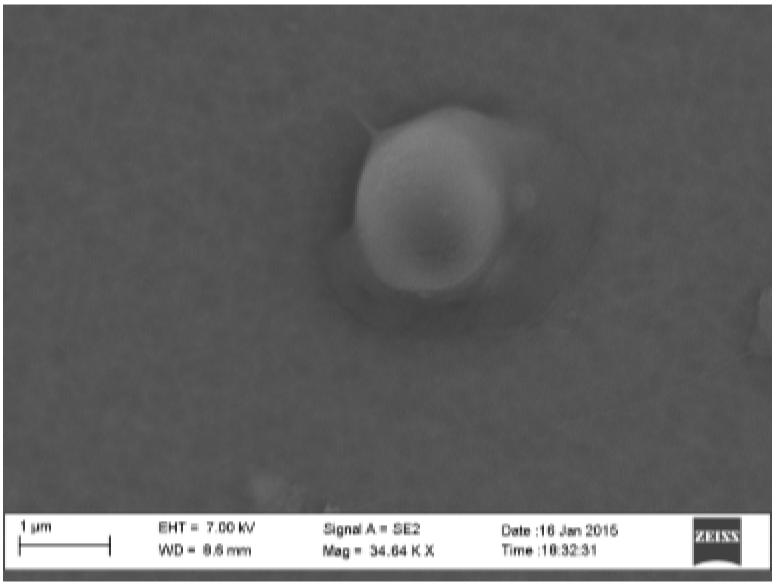


Figure S3. SEM image of an intact *S. aureus* cell on a control (Silicon wafer) surface

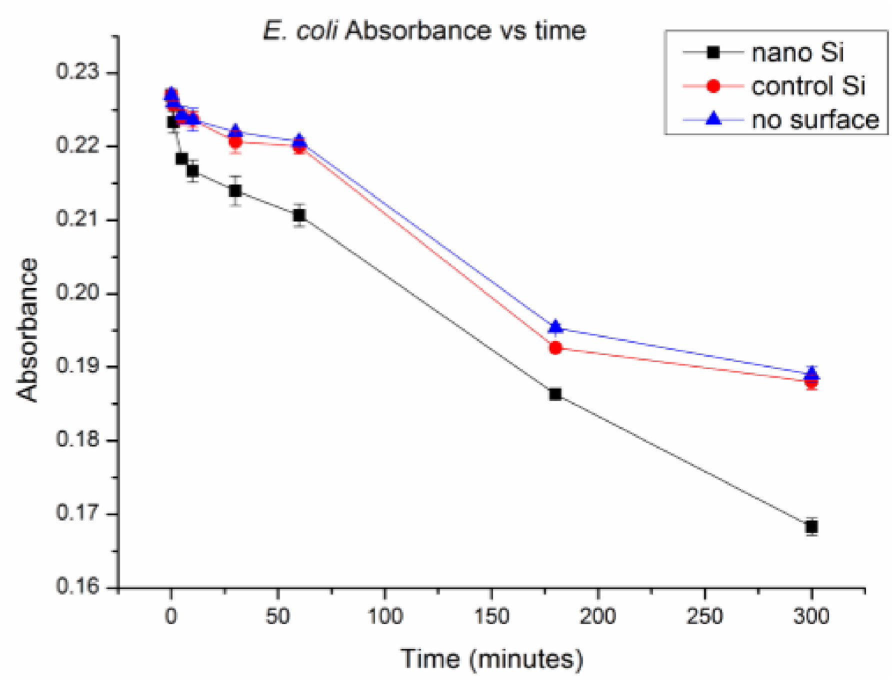


Figure S4. The absorbance values of the *E. coli* cells against the discrete incubation time intervals on the 0.5 cm × 0.5 cm of the controls and nanostructured surfaces. The surfaces were kept in 24-well plates where cells were present in 1 mL of 25 mM PBS solution and the OD measurements were taken after various time intervals.

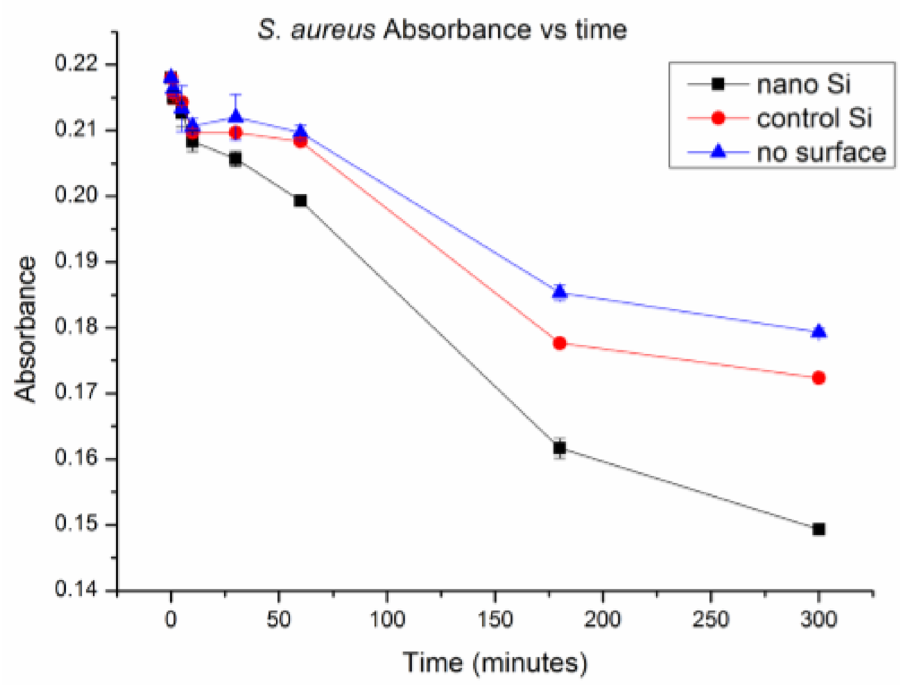


Figure S5. The absorbance values of the *S. aureus* cells against the discrete incubation time intervals on the 0.5 cm × 0.5 cm of the controls and nanostructured surfaces. The surfaces were kept in 24-well plates where cells were present in 1 mL of 25 mM PBS solution and the OD measurements were taken after various time intervals.

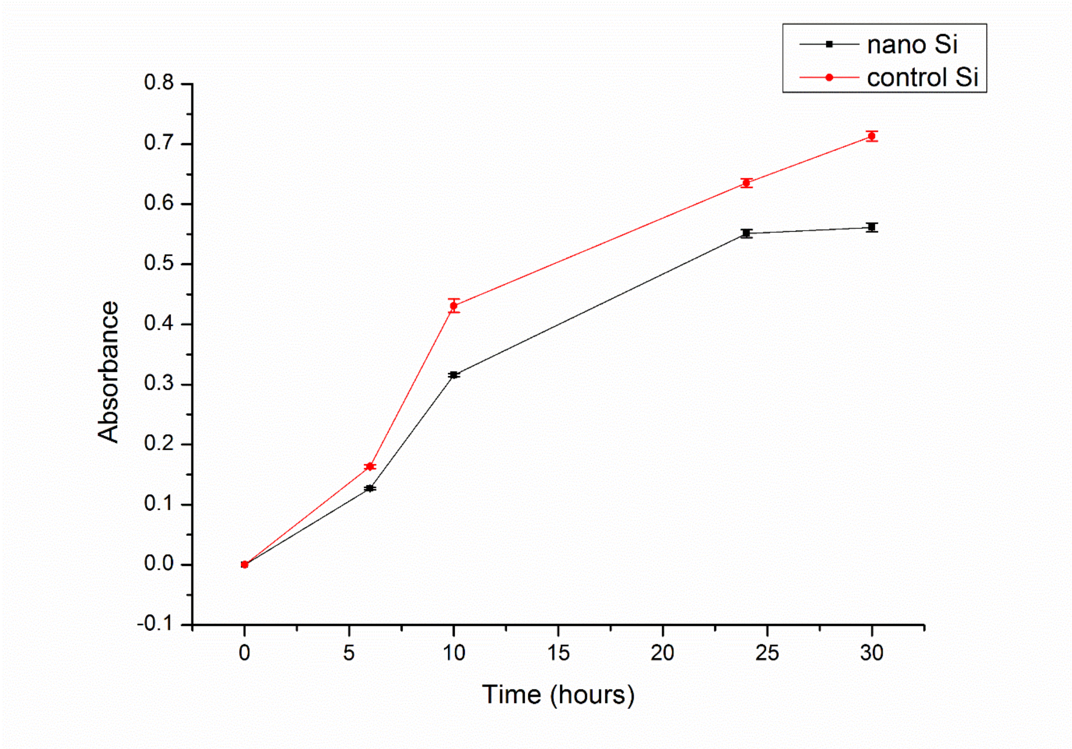


Figure S6. Absorbance vs time curve of the *E. coli* cells on the control and nanostructured surface in a growing nutrient media.

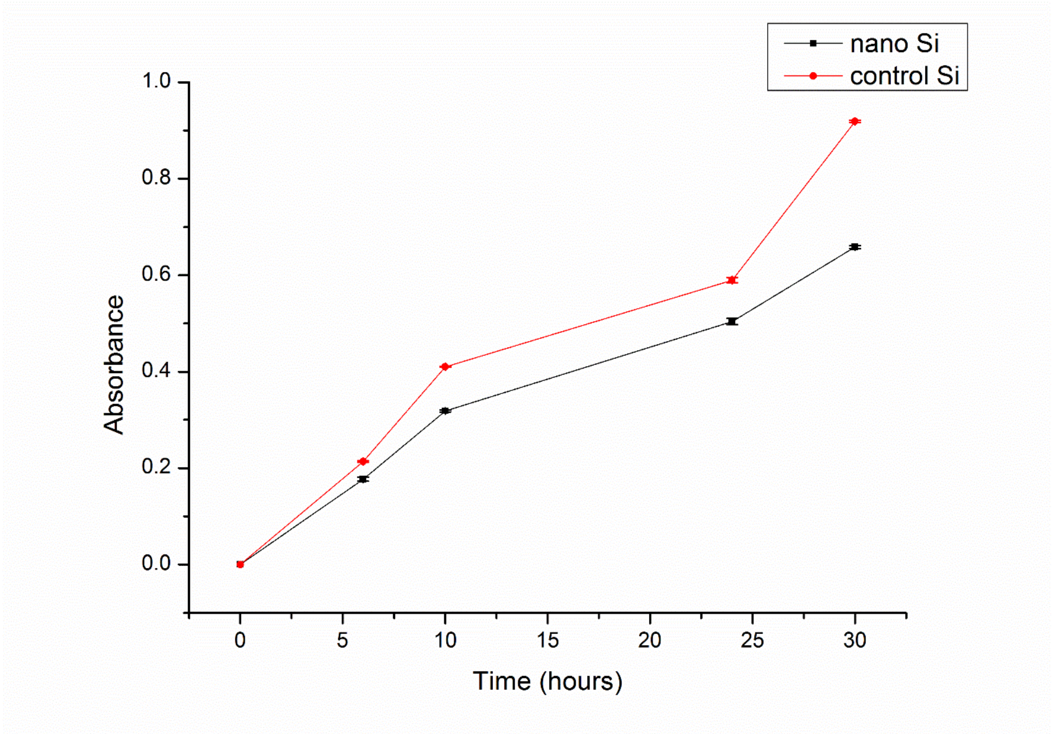


Figure S7. Absorbance vs time curve of the *S. aureus* cells on the control and nanostructured surface in a growing nutrient media.

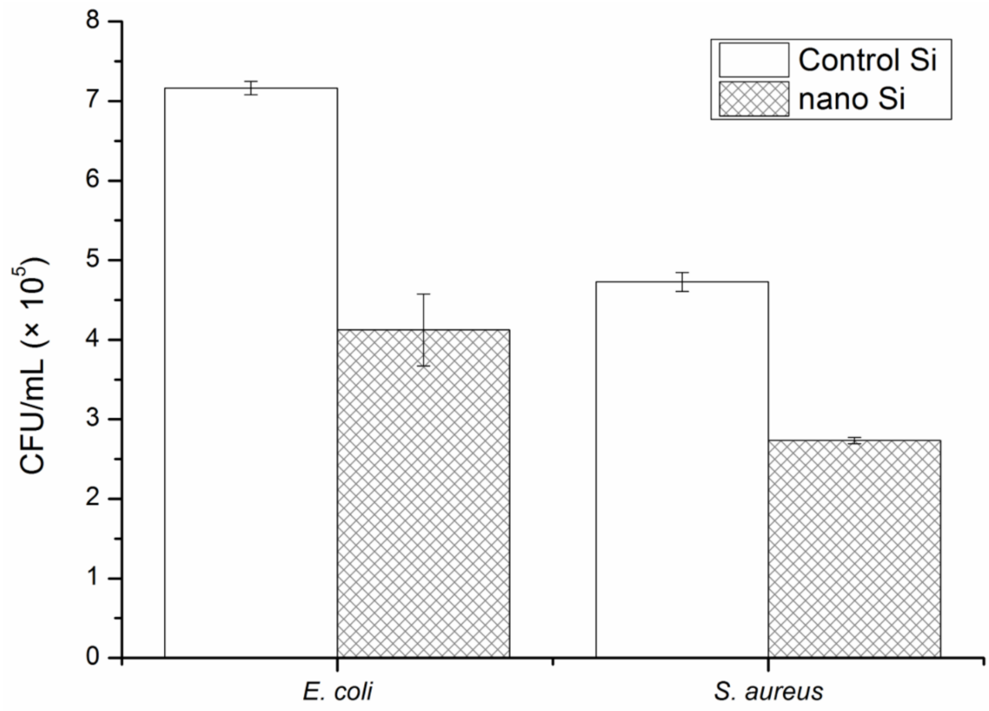


Figure S8. The colony forming units per mL of the two strains were measured after 1 hour incubation on control and nanostructured surfaces.

Table S1. Distribution of elements obtained from EDX spectra of the nanostructured silicon surface.

| Element | Weight % | Atomic % |
|---|---|---|
| C K | 20.68 | 35.71 |
| O K | 1.42 | 1.84 |
| F K | 13.90 | 15.17 |
| Si K | 64.00 | 47.27 |

Table S2. Distribution of elements obtained from EDX spectra of the control surface.

| Element | Weight % | Atomic % |
|---|---|---|
| C K | 12.73 | 25.44 |
| Si K | 87.27 | 74.56 |

Video S1. The low adhesive nature of the super surface is visible as the water droplet bounces and moves off the surface.
